# Micromechanics of emergent patterns in plastic flows

**DOI:** 10.1038/srep02728

**Published:** 2013-09-23

**Authors:** Santidan Biswas, Martin Grant, Indradev Samajdar, Arunansu Haldar, Anirban Sain

**Affiliations:** 1 Department of Physics, Indian Institute of Technology, Bombay, Powai, Mumbai-400 076, India; 2 Physics Department, McGill University, 3600 rue University, Montréal, Québec, Canada H3A 2T8; 3 Department of Metallurgical Engineering and Material Science, Indian Institute of Technology, Bombay, Powai, Mumbai-400 076, India; 4 Product Research Group, Research and Development Division, Tata Steel Limited, Jamshedpur 83100, India

## Abstract

Crystalline solids undergo plastic deformation and subsequently flow when subjected to stresses beyond their elastic limit. In nature most crystalline solids exist in polycrystalline form. Simulating plastic flows in polycrystalline solids has wide ranging applications, from material processing to understanding intermittency of earthquake dynamics. Using phase field crystal (PFC) model we show that in sheared polycrystalline solids the atomic displacement field shows spatio-temporal heterogeneity spanning over several orders of length and time scales, similar to that in amorphous solids. The displacement field also exhibits localized quadrupolar patterns, characteristic of two dislocations of the opposite sign approaching each other. This is a signature of crystallinity at microscopic scale. Polycrystals being halfway between single crystals and amorphous solids, in terms of the degree of structural order, descriptions of solid mechanics at two widely different scales, namely continuum plastic flow and discrete dislocation dynamics turns out to be necessary here.

Plastic flow is the continuum description of atomic displacements in a crystalline solid. It involves a hierarchy of activities at a wide range of length and time scales. While individual dislocations move (glide/climb) at the nanometer scale, cooperative movement of large number of dislocations causes grain boundaries to move at micron scales, ultimately leading to macroscopic response of the solid to applied stresses. The biggest challenge in the theoretical description of plastic flow is to bridge phenomena across these wide range of length and time scales. In spite of our lack of theoretical understanding, polycrystals, which are probably the most commonly occurring form of solids around us (metals and alloys, for example) are routinely processed in the industry^1^ using phenomenological protocols. An efficient approach towards bridging across scales is to use a Molecular dynamics (MD) simulation to extract parameters from a small system consisting of one or few dislocations, and then use the parameters for constructing a coarse-grained phenomenological description at the next higher scale. Driven by this philosophy here we study plastic flow in a micro-scale polycrystal consisting of few thousand atoms, a system appropriate for studying interplay between dislocations and grains. We subject a two dimensional (2D) polycrystalline sample to a constant strain rate^2^, by confining it between two infinite parallel plates (located at *y* = 0 and *y* = 2*H*) and moving them in opposite directions at constant speeds, 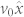 and 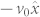, respectively as shown in Fig. 1 (see *methods*). Most simulation studies employ quasi-static strain, whether it is for amorphous solids where relaxational dynamics near equilibrium is studied^3^,^4^,^5^, or for crystalline solids where onset of plasticity mediated by dislocations is of interest^6^. Here we focus on the non-equilibrium steady state of a sheared polycrystal subjected to finite strain-rate *v*_0_/*H*. Finite strain rate ensures a continuous injection of dislocations through the boundaries. These dislocations interact with the dislocations in the bulk, the free ones as well as the ones bound to the grain boundaries, and some of them also escape through the boundaries, thereby establishing a steady state dislocation density in the sample.

The velocity field in sheared polycrystals shows striking similarity with that in amorphous material. Polycrystals exhibit heterogeneous distributions of velocity, vorticity and particle displacement which can be attributed to the existence of large number of grains and dislocations. MD simulations of sheared amorphous material, subjected to quasi-static strain rates^3^,^4^,^5^, have demonstrated that plastic displacements give rise to large scale vortices and few isolated, active spots. These spots generate quadrupolar displacement pattern around them and have been variously called STZs^7^ or elementary, plastic events^4^. Such localized spots are seen in sheared polycrystals as well (circled in our Fig. 2a). In terms of particle motion such a quadrupolar displacement pattern corresponds to emergence of an unstable saddle point. One stable and another unstable axes pass through such a point and particles move towards and away, respectively, from the point along these axes. While for amorphous materials it is not understood how such singular points are created in the interior of the system, in polycrystalline material generation of such saddle structures can be explained through the dynamics of the underlying dislocations, as we explain later. Similar saddle pattern can be generated by the dislocation motion in a strained single crystal also, near the onset of plasticity^6^. This implies that microscopic discreteness has to be properly accounted for in a coarse grained continuum description of the flow fields in a polycrystal. Picard et al^8^ used continuum theory to study the effect of a localized shear strain 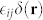, in a 2D viscoelastic medium (only the non-diagonal elements of 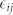 were assumed to be nonzero and equal). They showed how, through elastic interaction, the localized shear strain can induce a long range strain field. However this strain field turned out to be octapolar in nature, i.e., having four positive and four negative lobes.

We employ the modified phase field crystal (MPFC) model^9^,^10^ to simulate the polycrystal. PFC and MPFC models have been very successful in reproducing phenomenology of grain-boundaries^11^, premelting transition^12^, dislocation motion^13^, liquid crystals^15^ and glassy dynamics^14^. PFC and MPFC has also been derived^16^,^17^ from microscopic density functional theory. The strength of the phenomenological PFC model^9^,^10^ is that we can study dynamics of solids at the microscopic (atomic) length scales but diffusive time scales, much longer than that accessible by molecular dynamics (MD) simulations. Also here dislocations are generated spontaneously without any ad hoc rules being imposed.

PFC model is based on a Landau-Ginzburg type free energy functional involving a conserved, scalar order parameter (OP) *ψ*(**r**, *t*) which follows^2^,^11^

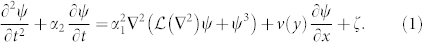
In this dimensionless form, the operator 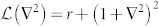, where *r* is the effective temperature. *ζ* is the conserved noise and strain is implemented^2^ by an imposed drift velocity field *v*(*y*) (see *methods*). The parameters *α*_1_, *α*_2_ control the time scale of the phonon modes propagating in the solid and the degree of their damping^10^.

## Results

**Figure 1:**
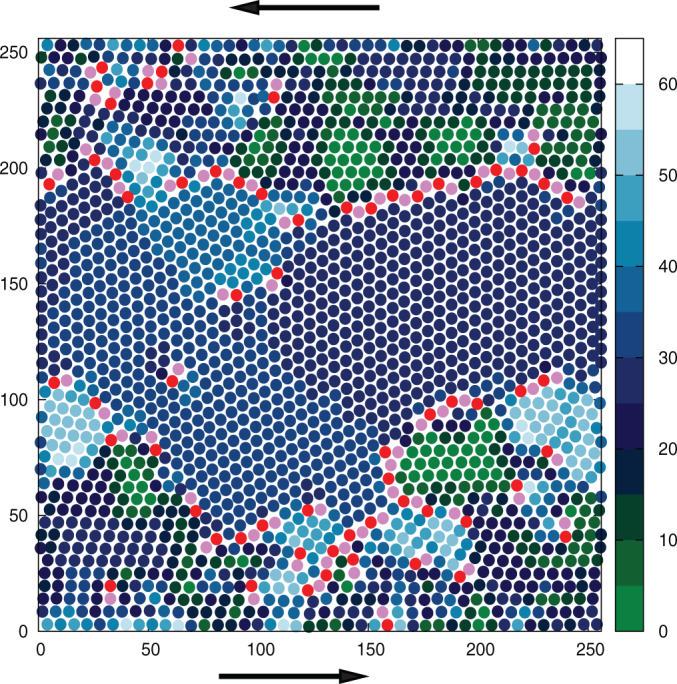
Sheared polycrystal: Spatial map of the local crystal orientation *θ*(r) with a range [0, *π*/3]. Dislocations, indicated by light/pink colors, decorate the grain boundaries with high misorientation. This is consistent with the Frank condition *n* ∝ sin *θ*^18^, which relates the line density of dislocations, *n* along a grain boundary with the corresponding misorientation angle *θ*.

In the PFC model, the particles (atoms) are identified^9^ as the minima of the OP field *ψ*(**r**, *t*). The grains in the 2D sheared polycrystal (see Fig. 1) has triangular symmetry with atomic co-ordination number six. Dislocations are identified^2^ by finding atoms with co-ordination number 5 or 7. The local crystallographic orientation, which distinguishes the grains, is given by an angle *θ*(**r**) ∈ [0, *π*/3] in our 2D geometry (see Fig. 1).

**Figure 2:**
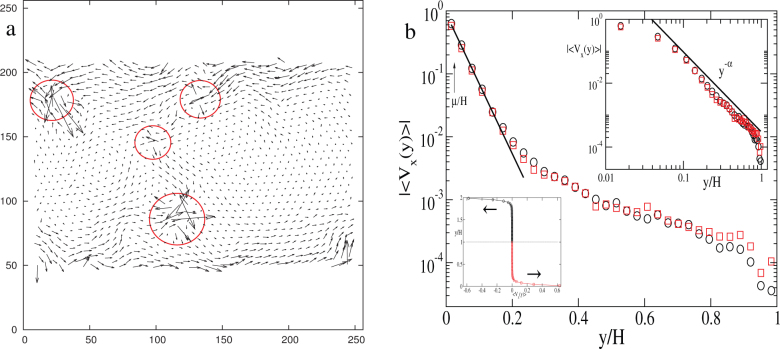
Velocity field of the sheared polycrystal. (a) Velocity pattern of the particles in the bulk showing vortices and isolated saddles (circled). Velocity vectors in the boundary layers are omitted as they are too large compared to that in the bulk (see lower inset of ‘b'). (b) Semi-log plot of |〈*v_x_*(*y*)〉| as a function of *y* (*y* increases towards the bulk). The velocity profiles for *y* > *H* (circles) and *y* < *H* (squares) are superimposed (after reflecting the *y* > *H* portion across the mid-plane, *y* = *H*) to show anti-symmetry. The solid line shows the imposed drift velocity *v*(*y*) = *v*_0_ exp(−*y*/*μ*). The same velocity data is plotted in the upper inset (log-log) to show approximate scaling 〈*v_x_*(*y*)〉 ~ *y*^−*α*^ in the bulk where 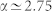, and in the lower inset (regular x-y) to highlight the separation between the boundary layer and the bulk.

In simple, viscous liquids the velocity profile is linear between two moving plates. However, in solids (amorphous/crystalline) that is not the case. In a polycrystalline solid presence of grains results in a strongly heterogeneous flow field. A grain resists motion till the accumulated strain crosses its elastic limit when it either rotates with respect to its neighboring grains or breaks up into smaller grains. Fig. 2a shows the detailed velocity map of the particles in the bulk. Despite the strong bias along 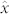 (the shear direction) the flow field in Fig. 2a shows significant motion along 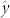, giving rise to characteristic vortical motion, and some irregular motion around few isolated points (circled in Fig. 2a). On an average (see Fig. 2b), the flow separates into a fast moving boundary layer and a slow moving bulk region (Fig. 2b). Further, in the bulk the flow is strongly heterogeneous: very slow in the interior of the grains and relatively faster at the grain boundaries. The slow large scale rotation which is visible on the right part of Fig. 2a can be identified with rotation of a grain. This will be discussed further later. The *y*–dependence of the average speed along *x*–direction is shown in Fig. 2b which shows an approximate power law behavior in the bulk and exponential behavior in the boundary layer. All these features have bearing on the heterogeneous displacement distribution discussed later.

**Figure 3:**
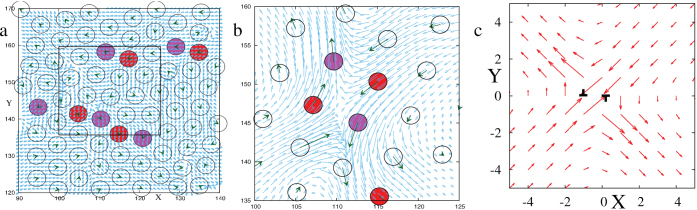
Plastic events originate from dislocation dynamics. (a) and (b) focus on a small region of the 256 × 256 lattice. The circles represent the atoms and the dark arrows on the circles represent their velocity vectors while the light/blue arrows represent the interpolated velocity field. The filled circles are the atoms with five or seven (pink or red) neighbors, indicating an edge dislocation. (a) shows initiation of a saddle as two oppositely ‘charged' dislocations approach. In (b) the boxed portion of ‘a' is zoomed at a later time, showing development of the saddle as the dislocations get close to generate a quadrupolar displacement pattern and annihilate subsequently (see Supplementary movie M1). Note that displacement is proportional to velocity for short time intervals. (c) shows the superposed displacement field of two edge dislocations (with opposite Burgers vector) calculated using Eq. (2). The parameters used are *b* = 1 and *ν* = 0.1. The dislocation positions are (0, 0) and (−1, 0).

At the isolated points with high activity, the motion of particles exhibit a quadrupolar pattern, essentially a saddle, in the displacement field of the particles. We show that such a pattern emerges from sideways approach of two oppositely ‘charged' edge dislocations towards each other. The sequence in Fig. 3a, b clearly shows time development of the displacement field leading towards a saddle as the dislocations approach each other. The saddle fades away after the dislocations annihilate (see Supplementary movie M1). Similar velocity pattern which appears as a saddle only at a scale larger than the minimum distance of approach between two oppositely ‘charged' dislocations, have been reported by Moretti et al., near the onset of plasticity in a single crystal, subjected to uniaxial, quasi-static strain. In their system the dislocation pairs were nucleated at a distance apart and they escaped to the boundary without forming a singular point, unlike our case where the dislocation pair approach arbitrarily close and eventually annihilate. Irrespective of these minor differences, which depends on the boundary condition, the strain protocol and the system size, saddles in polycrystals emerge from its underlying crystallinity at small scale.

If we assume that the strain field created by the dislocations is quasistatic then the quadrupolar displacement pattern around a oppositely ‘charged' dislocation pair can be quantitatively understood. This quasistatic approximation is plausible since the bulk is highly screened by the boundary layers and therefore has a very slow dynamics. Now it is known that the equilibrium strain field of an edge dislocation generates a displacement dipole^19^ where the positive and the negative lobes are oriented along the axis connecting the atoms with coordination numbers 5 and 7. Such a 5 – 7 pair is like a charge dipole and sideways arrangement of two such pairs form a quadrupole. Essentially these two dislocations have opposite burgers vectors 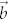 and 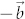 along one of the symmetry axes of the crystal. In comparison, dislocations with the same burgers vector can arrange in a linear fashion “..5-7-5-7-5-7..” to form a dislocation wall (see Fig. 1) which is rather stable. These walls are equivalent to high angle grain boundaries (Fig. 1). The quadrupolar structure discussed above can be quantitatively established by superimposing the elastic displacement fields of two dislocations located close by. Fig. 3 shows the resultant field from two dislocations located at 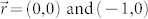, where displacement field 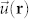 due to a dislocation at the origin is given by^19^

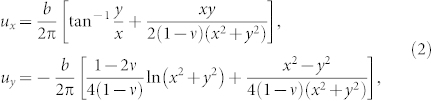
where *ν* is the Poisson ratio and tan^−1^ 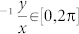.

**Figure 4:**
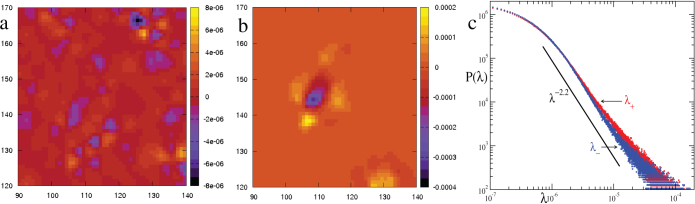
Spatial distribution of vortical and extensional zones. (a) Okubo-Weiss field *λ*(*x*, *y*) in the bulk, corresponding to the velocity fields a and b of Fig. (b) clearly shows the prominent saddle (dark/violet shade) and the surrounding vorticity field (light/yellow shade) arranged in a quadrupolar shape. Note that, in (a) also many saddles (violet) are visible but their intensity is two order of magnitude weaker than that in (b). (c) Log-log plot of the distribution of the Okubo-Weiss field. We show separate distribution functions for the positive (vorticity) and negative (saddle) values of *λ*, indicated by *λ*_+_ and *λ*_−_ respectively (*λ*_−_ = |*λ*| when *λ* < 0). The distributions are nearly symmetric and has power law regime, indicating hierarchy in the strength of activity.

In order to study the spatial distribution of vorticity and saddles in our 2D plastic flow we employ a quantitative measure used in fluid turbulence^20^. For 2D inviscid, incompressible flows the Okubo-Weiss parameter is defined as 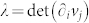. This is an invariant of the flow and can be recast as 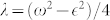. Here 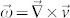 is the vorticity vector and 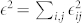, where 
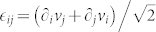
 is the strain rate tensor. Even in viscous flows *λ* turns out to be an useful measure^21^ and regions with vortices have *λ* > 0, while the strain dominated regions have *λ* < 0. Note that a saddle corresponds to stretching in one direction and compression in the orthogonal direction, essentially creating a strain dominated region. Our system is not strictly incompressible, but in terms of total particle number, the fluctuation is less than 1% (less than 20 in 2000). For computing *λ* we interpolated the particle velocities onto a finer square grid. Fig. 4a, b shows a spatial map of the Okubo-Weiss field *λ*(**r**) corresponding to the velocity fields in Fig. 3a, b. We also compute the probability distribution function (PDF) of *λ* which is shown in Fig. 4c. It shows that, spatially, strain dominated regions occur almost as frequently as vortex dominated regions. Further, approximate power law scaling of *P*(*λ*) indicates that these regions are organized in a scale free hierarchical structure.

**Figure 5:**
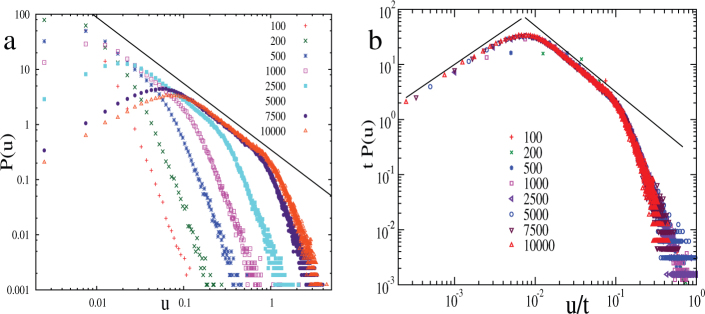
Displacement distribution exhibiting spatio-temporal heterogeneity. (a) Distribution *P*(*u*) of displacement magnitudes(*u*) of the particles in the bulk, collected after different time intervals *t* (shown in arbitrary units in the figure). (b) shows collapse of the plots shown in ‘a', into a single master curve, after rescaling of *u* by *t*, and *P*(*u*) appropriately. The collapse occurs since the average *x*–displacement 〈*u_x_*〉 = 〈*v_x_*〉*t* dominates *u*. Origin of the scaling behaviors, fitted by the solid lines *P*(*u*) ∝ *u* and ∝ *u*^−1.2^), are explained in the text.

Finally we report intriguing power laws in the probability distribution function (PDF) of the particle displacements 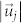, in the bulk (excluding the boundary region where 〈*v_x_*(*y*)〉 is large). Here *j* is the particle index. The displacements 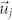 after large time intervals show characteristic patterns, around the plastic events (figure not shown here). The PDF of 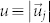 is shown in Fig. 5a, which, at large time intervals *t*, shows two clear power law regimes. Rescaling *u* with *t* (and also *P*(*u*) appropriately) the PDFs' collapse nicely (see Fig. 5b), although the PDFs' for short *t* do not have any power law regime and is dominated by fluctuations.

## Discussion

The displacement distribution turns out to be an excellent marker for spatio-temporal heterogeneity. The different scaling regimes of *P*(*u*) can be connected to distinct kind of particle motion in the bulk of the sheared polycrystal. *P*(*u*)*du* is the fraction of particles undergoing particular type of motion and is therefore approximately proportional to the area fraction occupied by these particles in a typical velocity map like Fig. 2a. For example, the displacements are small at the core of the large grains where motion is vortical. Assuming a slow rotational speed *ω*_0_, the displacement *u*, for 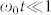, is *u* ~ *ω*_0_*rt*, where the radius *r* is measured with respect to the center of the grain. Thus *P*(*u*)*du* ∝ *dA* = 2*πrdr* and using *u* ~ *ω*_0_*rt*, we get 
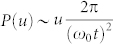
. Consequently a time independent collapse occurs in the *P*(*u*)*t* versus *u*/*t* plot (Fig. 5b). At larger displacements the scaling *P*(*u*) ~ *u*^−1.2^ is dominated by 〈*v_x_*〉 which approximately scales as 〈*v_x_*(*y*)〉 ~ *y*^−*α*^ in the bulk (upper inset of Fig. 2b) and 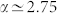. Here, *P*(*u*)*du* ∝ *dA* = *Ldy* (where *L* is the box length) and using *u* ~ *y*^−*α*^*t*, we get 
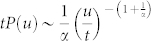
, again a *t* independent collapse. But 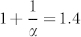; this small mismatch (with numerical value −1.2) depends on the amount of the boundary layer that we exclude while computing *P*(*u*), in particular the numerical exponent goes to −1.4 when we exclude more compared to that in Fig. 2a. This is consistent with the observation that the boundary layer where 〈*v_x_*(*y*)〉 ~ *e*^−*y*/*μ*^, contribute *u*^−1^ scaling, and therefore reduces the effective exponent. Further we verified that the high displacement tail comes purely from the active spots.

**Figure 6:**
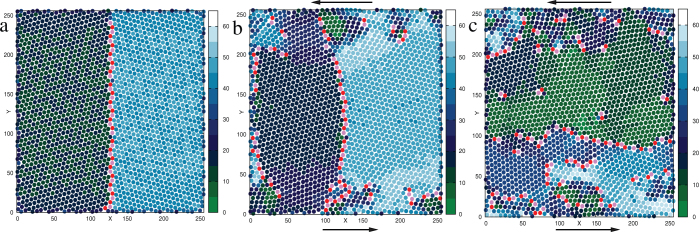
Grain rotation in sheared bicrystal. (a) shows an equilibrated 2-D bicrystal having misorientation of 30° at the high angle grain boundary. Local crystal orientation field is indicated in the color bar. Dislocations (5–7 pairs in pink and red) arrange vertically along the grain boundary. Dislocations at the second grain boundary located at the vertical edges of the box (due to periodic boundary condition) are not shown here. b) shows rotation and distortion of the grain boundary once shear is applied, by imposing a constant strain rate. (c) shows a polycrystalline grain structure which results after sufficient time.

It is important to distinguish between the local vortical motion which is initiated when two dislocations of opposite charge approach each other (see Fig. 2a, 3b and 4b) and the slow rotation of the large grains which leads to the linear scaling regime in Fig. 5. Transient local motion of the first kind, induced by defect motion, has been previously observed by Moretti et al.^6^ in their MD simulation where oppositely charged dislocation pairs were nucleated near the onset of plasticity under quasistatic, uniaxial, compressional strain. Grain rotation, on the other hand involves a collective motion of many dislocations as the grain boundary rotates in response to the strain gradient which develops in the interior due to the external strain rate applied at the boundaries. In Fig. 6 we show how the grain boundary rotate and subsequently distort in a bicrystal in response to globally applied strain rate. At finite strain rate (*v*_0_ = 0.07 in Fig. 6) a polycrystalline, nonequilibrium state is achieved after some time during which the initial grains have rotated and distorted significantly. The formation of polycrystalline structure in fact offers a mechanism for dislocation storage, which is absent in single crystals near the onset of plasticity with only few dislocations (see Ref. 6, 13).

In summary, we have shown that the plastic flow in sheared polycrystals show strong spatio-temporal heterogeneity which manifests as three distinct regimes in the displacement distribution of the particles. Here, presence of grains of different sizes renders the motion more heterogenous, as compared to amorphous solids. Furthermore, the elementary plastic events of the flow field can be explained in terms of the underlying dislocation dynamics. We imagine that such a diverse flow field could be experimentally observed in sheared colloids. Collective motion of particles, forming strings or showing caged diffusion, have already been observed^22^ at grain boundaries of colloidal polycrystals through particle tracking experiments.

## Methods

The results presented here are from simulations on a square grid of size 256 × 256. No qualitative difference was found for a bigger grid (up to 1024 × 1024), except that the data could be much better averaged for the smaller grid due to shorter run time. The conserved, Gaussian noise in Eq.1 is delta correlated in space and time and follows^11^, 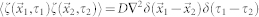, 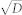 being the noise amplitude. Eq.1 is time evolved in the Fourier space using integrating factor method. In our shearing scheme^2^ instead of moving only the top and the bottom surfaces of the solid, a drift velocity profile 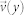 which decays exponentially away from the boundary surfaces (towards the bulk) is imposed on the solid: 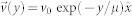 for 0 < *y* < *H* and 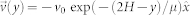 for *H* < *y* < 2*H*. We verified that keeping *μ* fixed if we increase the height 2*H* ( = 256 → 512 → 1024) of the sample, the thickness of the boundary layer (defined by the point where 〈*v_x_*〉 deviates from the ~ *e*^−*y*/*μ*^ scaling) decreases with respect to 2*H*. We applied periodic boundary condition (with box size *L*) in the horizontal direction. The values used for the simulations are *α*_1_ = 1, *α*_2_ = 1, *r* = −0.5, 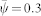, *D* = 1, *v*_0_ = 0.45, *μ* = 40, *dx* = *dy* = *π*/2 and *dt* = 0.025.

## Author Contributions

S.B. and A.S. designed the project, wrote the manuscript and prepared all the figures. M.G., I.S. and A.H. contributed in designing the project. All authors reviewed the manuscript.

## Supplementary Material

Supplementary Information Supplementary Movie 1
